# Nanostructured Titanium-10 wt% 45S5 Bioglass-Ag Composite Foams for Medical Applications

**DOI:** 10.3390/ma8041398

**Published:** 2015-03-25

**Authors:** Karolina Jurczyk, Grzegorz Adamek, Marcelina M. Kubicka, Jaroslaw Jakubowicz, Mieczyslaw Jurczyk

**Affiliations:** 1 Department of Conservative Dentistry and Periodontology, Poznan University of Medical Sciences, Bukowska 70 St., 60-812 Poznan, Poland; E-Mail: karolajur@gmail.com; 2 Institute of Materials Science and Engineering, Poznan University of Technology, Jana Pawla II 24 St., 61-138 Poznan, Poland; E-Mails: grzegorz.adamek@put.poznan.pl (G.A.); jaroslaw.jakubowicz@put.poznan.pl (J.J.); 3 Department of Genetics and Pharmaceutical Microbiology, Faculty of Pharmacy, Poznan University of Medical Sciences, Swiecickiego 4 St., 60-781 Poznan, Poland; E-Mail: rufin1985@interia.pl

**Keywords:** biomaterials, ceramic-metal composite, powder metallurgy, *Staphylococcus aureus*

## Abstract

The article presents an investigation on the effectiveness of nanostructured titanium-10 wt% 45S5 Bioglass-1 wt% Ag composite foams as a novel class of antibacterial materials for medical applications. The Ti-based composite foams were prepared by the combination of mechanical alloying and a “space-holder” sintering process. In the first step, the Ti-10 wt% 45S5 Bioglass-1 wt% Ag powder synthesized by mechanical alloying and annealing mixed with 1.0 mm diameter of saccharose crystals was finally compacted in the form of pellets. In the next step, the saccharose crystals were dissolved in water, leaving open spaces surrounded by metallic-bioceramic scaffold. The sintering of the scaffold leads to foam formation. It was found that 1:1 Ti-10 wt% 45S5 Bioglass-1 wt% Ag/sugar ratio leads to porosities of about 70% with pore diameter of about 0.3–1.1 mm. The microstructure, corrosion resistance in Ringer’s solution of the produced foams were investigated. The value of the compression strength for the Ti-10 wt% 45S5 Bioglass-1 wt% Ag foam with 70% porosity was 1.5 MPa and the Young’s modulus was 34 MPa. Silver modified Ti-10 wt% 45S5 Bioglass composites possess excellent antibacterial activities against *Staphylococcus aureus*. Porous Ti-10 wt% 45S5 Bioglass-1 wt% foam could be a possible candidate for medical implants applications.

## 1. Introduction

Metal foam is a class of interesting material that can exhibit a unique combination of physical, chemical and mechanical properties [1,2]. In particular, because it is lightweight and low Young modulus, titanium-based foam is drawing much attention in medical applications from the viewpoint of bone ingrowth promotion and induction of prosthesis stabilization.

Preparation of porous metals can be obtained by several methods, which have been described in multiple studies [1,3,4,5,6,7,8,9,10,11,12,13]. Aiming at bone ingrowth, long-term stability and load-bearing, the porous titanium should represent the following features [8]: (i) high porosity and interconnected pore structure for sufficient space enabling attachment and proliferation of new bone tissue and the transport of body fluids; (ii) within a critical pore size range, usually 150–500 µm; and (iii) appropriate mechanical properties to surround bone tissue for load-bearing and transforming.

Current research focuses on improving the mechanical performance and biocompatibility of metal-based systems through changes in alloy composition, microstructure and surface treatments [4,14,15,16]. In the case of titanium, a lot of attention is paid to enhance the strength characteristics of commercial purity grades in order to avoid potential biotoxicity of alloying elements, especially in dental implants [17].

Improvement of the physicochemical and mechanical performance of Ti-based implant materials can be achieved through microstructure control, the top-down approaches known as severe plastic deformation (SPD) and mechanical alloying (MA) techniques [15,18]. Recent studies showed clearly that nanostructuring of titanium can considerably improve not only the mechanical properties, but also the biocompatibility [5,14,15,16,18]. Nanostructured materials can exhibit enhanced mechanical, biological, and chemical properties compared with their conventional counterparts [16]. Over the past few years, application of nanoscale materials has become very popular in medicine.

An alternative method for changing the mechanical, chemical and biological properties of Ti and Ti-based alloys is the production of a composite, which represents the favorable mechanical properties of titanium and excellent biocompatibility and bioactivity of ceramics [15,19,20,21]. The main ceramics, used in medicine are hydroxyapatite (HA, Ca_10_(PO_4_)_6_(OH)_2_), silica (SiO_2_) or 45S5 Bioglass [22]. The ceramic coating on the titanium improves the surface bioactivity, but often flakes off as a result of poor ceramic/metal interface bonding, which may cause a post-operative failure. Therefore, the nanocomposite materials containing metal and bioceramic as a reinforced phase are promising alternatives to conventional materials, because they can potentially be designed to match the properties of bone tissue in order to enhance patient’s quality of life.

The common technique of metal foam preparation is sintering with a space holder [8,11]. All range of space holder materials are used, such as ammonium hydrogen carbonate, carbamide, magnesium, sodium chloride, sodium fluoride and titanium dihydride. Recently, new method of titanium foam preparation with saccharose crystals (C_12_H_22_O_11_) as space-holder particles was presented [23]. Other metal foam preparation techniques include polymer replication [8], controlled expansion of entrapped argon gas [11], freeze casting [24], laser processing [25], and rapid prototype methods [12,13].

Several studies have focused on the design of foams with optimized architecture to fulfill physico-chemical, mechanical as well as regeneration requirements [1,7,8,11]. For example, the compressive plateau stress and the Young’s modulus of the TiZr foam with relative densities of approximately 0.3 were 78.4 MPa and 15.3 GPa, respectively [3]. These foams displayed an interconnected porous structure resembling bone and the pore size ranged from 200 to 500 µm. Both the porous structure and the mechanical properties of the TiZr foam were very close to those of natural bone.

The aim of our research is to develop a new generation of titanium-bioceramic nanocomposites by producing the porous structure with a strictly specified chemical and phase compositions, porosity and surface morphology, which will adhere well to the substrate, show high hardness, high resistance to biological corrosion and good biocompatibility with human tissues [16]. We present a novel route for synthesis of a new class of Ti-45S5 Bioglass foams for use in biomedical implant applications. Porous implants have lower density than respective bulk ones, however good mechanical strength is provided by bulk substrate. In this paper, titanium-10 wt% 45S5 Bioglass-1 wt% Ag nanocomposite foams with porosities of about 70% and pore diameter of about 1.0 mm were developed. The influence of the microstructure on the mechanical and corrosion properties was studied. Finally, *in vitro* cytocompatibility test and antibacterial activity against *Staphylococcus aureus* were conducted.

## 2. Materials and Methods

**Figure 1:**
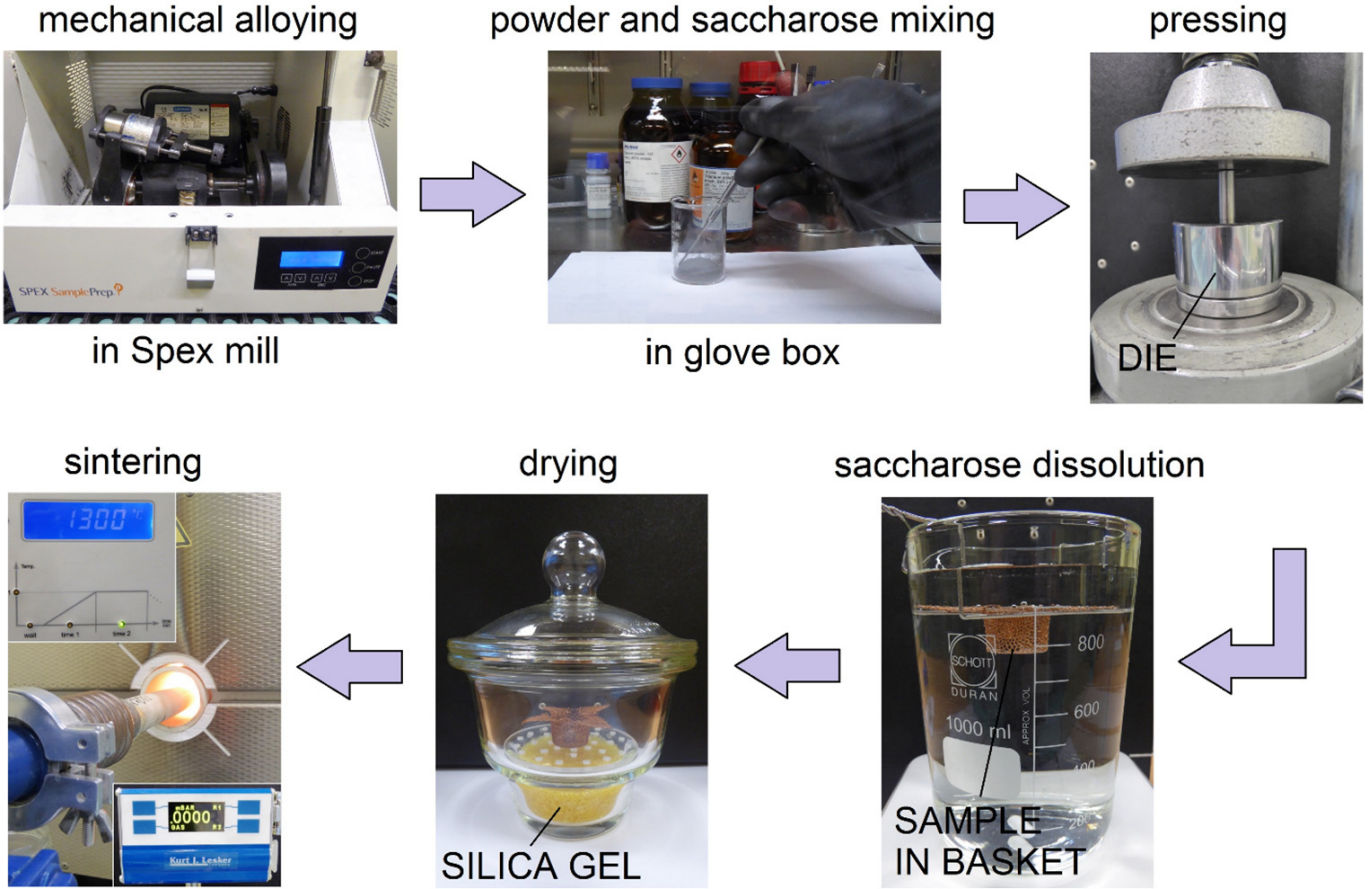
Overview of titanium-45S5 Bioglass foam synthesis.

The Ti-10 wt% 45S5 Bioglass-1 wt% Ag composite foams (denoted by Ti-Bioglass-Ag) were prepared by mechanical alloying (MA) and powder metallurgy process. Mechanical alloying was performed under argon atmosphere using a SPEX 8000 Mixer Mill. The mixed powders were first blended by mechanical alloying process with hard steel balls for 15 h (balls-to-powder ratio was 15:1). In the next step, mixture of amorphous Ti-based powders and table sugar crystals (1 mm, Pfeifer & Langen, Gostyn, Poland) (chemical name: saccharose) have been used for scaffolds preparation. We applied similar procedure in our previous work [23] for foams preparation from pure microcrystalline titanium. In our current study, we use amorphous/nanocrystalline powder, which is highly flammable. For safety reasons the mixing stage was done in a glove box with Ar atmosphere. The 5.0 g of Ti-Bioglass-Ag and 4.18 g of sugar particles were manually mixed together for 1 min using a stainless steel spatula in a 100 mL glass beaker. To facilitate particle bending, 2 g of ethanol was added into the mixture. The weight of all substances was measured using precision balance (0.001 g repeatability). The small amount of ethanol and short mixing time does not negatively effect on the sugar crystals degradation. In the next step, the mixture was portioned, placed into the matrix and uniaxially pressed at a pressure of 1000 MPa. The green compacts were 8 mm diameter and 5 mm high. In the next stage, the green compacts were placed into a glass beaker filled with 1 liter of water at a temperature of 20 °C. A magnetic stirring with speed of 250 rpm resulted in sugar removal from the green compacts neighborhood. After sugar dissolution stage (60 min), the porous green Ti-Bioglass-Ag compacts were dried for 24 h at RT in desiccator filled with balls strongly absorbing moisture. Then in the final stage, the green compacts were heated over 5 h to 1300 °C and kept at this temperature for 2 h for particle sintering. After that, the sinters were slowly cooled down to room temperature together with the furnace. The sintering was done at 10^−2^ Pa vacuum in an alumina tube (McDanel Adv. Ceram. Techn., Beaver Falls, PA, USA). Porous Ti-Bioglass-Ag composites with porosities of about 70% were fabricated (Figure 1).

For all tests, the porous Ti-based foams as well as microcrystalline Ti (Grade-2, Alfa Aesar; prepared from commercial titanium cylindrical 8 mm diameter rod by slicing into the disks with the thickness of 3 mm) were polished with 2500 SiC paper in water and ultrasonically rinsed with acetone. The phase constitution of Ti-based foams was analyzed by X-ray diffraction with Cu Kα_1_ radiation (Panalytical, Empyrean model, Almelo, Netherlands). The average crystallite sizes *d* were estimated by Scherrer method: β = 0.9 λ/*d* cos θ, where β is the full-width at half maximum intensity of a Bragg reflection excluding instrumental broadening, θ the Bragg angle and λ the wavelength of the X-ray radiation. Scanning electron microscope (SEM, VEGA 5135 Tescan, Brno, Czech Republic) with energy dispersive spectrometer (EDS, PTG Prison Avalon, Princeton Gamma Tech., Princeton, NY, USA) was used to characterize the chemical composition of the prepared foams.

The Vega Tescan SEM and Carl Zeiss Metrotom 800 computed tomography (CT, Carl Zeiss, Oberkochen, Germany) were applied for the foams pore and scaffold morphology investigation. Pore size distributions for foam materials were analyzed using the CT scans. The density of the sintered samples was determined by Archimedes method. The porosity of the foams was calculated by the formula *P* = (1 − ρ/ρ_th_) × 100%, where ρ and ρ_th_ are the density of the porous material and its corresponding theoretical density, respectively. Compressive strength was measured at RT using 4483 Instron mechanical testing machine, with strain rate 0.001^−1^.

The Solartron 1285 potentiostat was applied for corrosion measurements. The corrosion resistance of different samples was measured in Ringer’s solution (NaCl, 9.0 g/L; KCl, 0.42 g/L; CaCl_2_, 0.48 g/L and NaHCO_3_, 0.2 g/L) applying potentiodynamic mode with scan rate 0.5 mV/s at temperature of 37 ± 1 °C, controlled by thermostat. The corrosion test was run in EG&G K0047 corrosion cell. The counter electrode consisted of two graphite rods, and a saturated calomel electrode (SCE, Hydromet, Gliwice, Poland) was used as the reference electrode. The surface area exposed to the electrolyte was 0.5 mm^2^. Polarization curves were obtained for each specimen. The corrosion potentials (*E*_C_) and corrosion current densities (*I*_C_) were estimated from the Tafel extrapolations of the corrosion curves, using CorrView software (Scribner Associates Inc., Southern Pines, NC, USA).

Normal Human Osteoblast (NHOst) cells from Cambrex (CC-2538, Walkersville, MD, USA) were used for the *in vitro* cytocompatibility tests in static conditions. The cells were cultured at a concentration of 5000 cells/well in 1 mL of culture medium on each disc at 37 °C in a 5% CO_2_ incubator for 24 h. Then, the cells were fixed with a 25% glutaraldehyde solution for 10 min and stained with a 10% Giemsa’s staining solution for 10 min. In order to perform scanning electron microscopy examination, the specimens were sputter-coated with gold.

In this study, the *S. aureus* (ATCC 6538) strain was assessed. Cultures of *S. aureus* were obtained from commercial sources (American Type Culture Collection), and an aliquot was inoculated into 5 mL of Tryptic Soy Broth (TSB) and incubated overnight. The resulting log-culture had a concentration of ~10^9^ CFU/mL (Colony Forming Unit/mL). The experimental samples (typical dimensions were *d* = 8 mm in diameter and *h* = 3 mm in height) were placed in 5-mL sterilized tubes followed by the addition of 0.5 mL of the bacterial suspension to TSB. The tubes were incubated at 37 °C with shaking at 200 rpm for 3 h. At the end of the incubation period, the samples were gently rinsed three times with PBS in order to eliminate the non-adherent bacteria. Then the samples were put into individual new tubes containing 5 mL of PBS each, and then subjected to sonication at a frequency of 20 kHz (Bandelin Sonoplus) for 5 min, followed by additional vortexing for 60 s to remove the adhering micro-organisms. The viable organisms in the buffer were quantified by plating serial dilutions on Typtic Soy Agar (TSA) plates. TSA agar plates were incubated at 37 °C and the colony-forming units were counted visually.

All experiments were repeated three times. The commercially available program Excel (Microsoft, Corporation, Redmond, WA, USA) and statistical software R version 3.0.1 (Foundation for Statistical Computing, Vienna, Austria) were used to determine whether any significant difference existed in bacterial number in the antibacterial experiments. Analysis of variance (ANOVA) followed by Tukey’s honest significant difference (HSD) test was performed on the bacterial counts. The statistical significance was defined as *p* < 0.05.

## 3. Results

**Figure 2:**
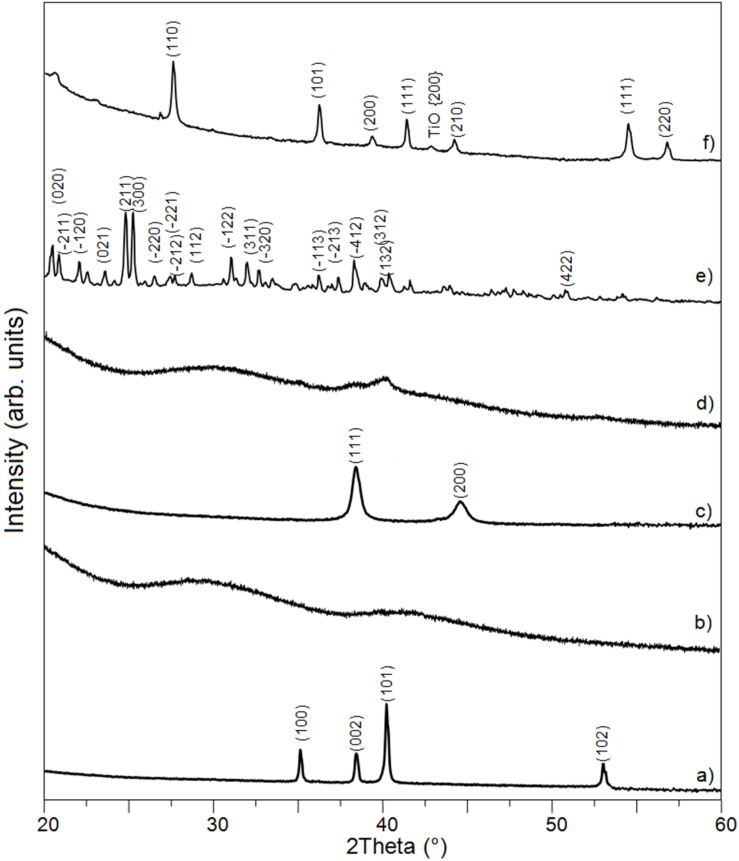
XRD spectra of Ti (**a**); 45S5 Bioglass (**b**) and silver (**c**) powders, their mixture after 15 h of mechanical alloying (**d**); saccharose crystals (C_12_H_22_O_11_)—the space-holder material (**e**) and Ti-10 wt% 45S5 Bioglass-1 wt% Ag nanocomposite foam with porosity of 70% after space holder removing and scaffold sintering in a vacuum of 10^−2^ mbar at 1300 °C for 2 h (**f**).

In the present work, the Ti-Bioglass-Ag composite foams with porosity of about 70% were successfully synthesized. The mixture of microcrystalline titanium (ICDD: 5-682), amorphous 45S5 Bioglass and microcrystalline silver (ICDD: 4-783) powders were blended and mechanically alloyed in argon atmosphere using a SPEX Mixer Mill (see Figure 2a–c). The microstructure that formed during MA consists of layers of the starting material. The lamellar structure is increasingly refined during further mechanical alloying. After 15 h of mechanical alloying, the precursor showed cleavage fracture morphology and inhomogeneous size distribution. The titanium and silver underwent MA for 15 h, decomposing mostly into an amorphous Ti-Bioglass-Ag phase and nanocrystalline Ti. According to the Scherrer method, the average crystallite size of obtained nanocrystalline Ti was in the range of 10 nm (Figure 2d).

**Figure 3:**
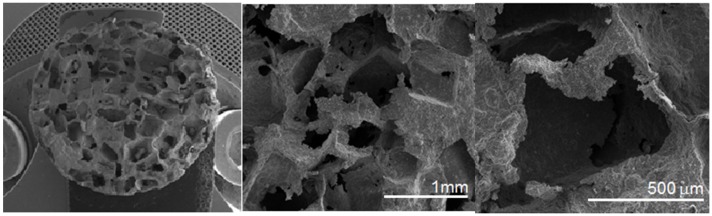
SEM pictures of the Ti-10 wt% 45S5 Bioglass-1 wt% Ag nanocomposite foam with porosity of 70% at different magnifications.

**Figure 4:**
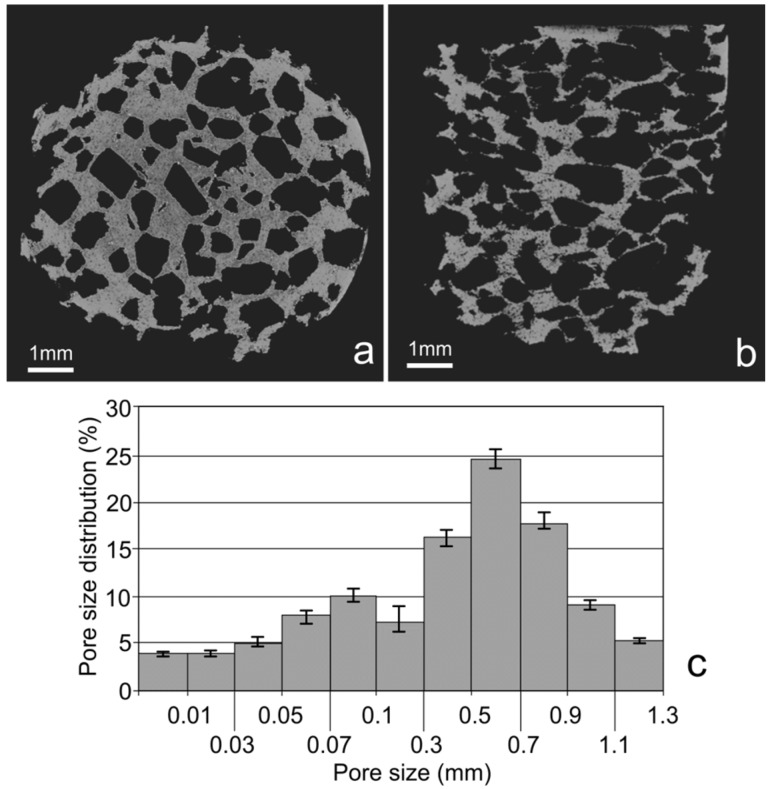
CT cross sections recorded at different sample position—top view (**a**); top-side view (**b**); and calculated pore size distribution (**c**).

In the next step, the mixture of amorphous Ti-Bioglass-Ag powders and table sugar crystals (see Figure 2e) was used for void metal foam preparation. Finally, the green scaffolds were sintered at 10^−2^ Pa vacuum at 1300 °C. Porous Ti-Bioglass-Ag composites with porosities of about 70% were fabricated (see Figure 2f). SEM and computed tomography pictures of the foams are shown on Figure 3 and Figure 4. Symmetrical distributed pores, rectangular in shape with straights walls, are present. The final shape of the pores is due to the shape of the initial symmetrical sugar crystals. The foam has a complex microstructured surface and exhibits wide cavities. Both SEM and CT detected formation of two types of pores in the foams. Larger macropores were developed by space holder sugar particles (0.3–1.1 mm) and smaller ones appeared between pressed and sintered Ti-45S5 Bioglass-Ag particles (5–100 µm diameter)—see Figure 4c. Moreover, the pores forming in the foam were mostly interconnected, which was detected with the aid of CT measurements.

XRD analysis of Ti-Bioglass-Ag foam with porosity of about 70% showed the presence of rutile (TiO_2_) with cell parameters *a* = 4.593 Å, *c* = 2.959 Å with a small content of TiO phase (Figure 2f). The formation of Na_2_Ca_2_Si_3_O_9_ as the main crystalline phase [26] with minor Na_2_CaSi_3_O_8_ phase [27] is achieved after thermal treatment of 45S5 Bioglass above 600 °C, although in this study the phases have not been detected due to small amount of Bioglass in titanium. For some authors, raising the temperature to 800 °C for long period leads to the development of calcium phosphate crystals [28]. However, the real composition of the crystals appearing during thermal treatment of Bioglass still remains unclear [29].

**Figure 5:**
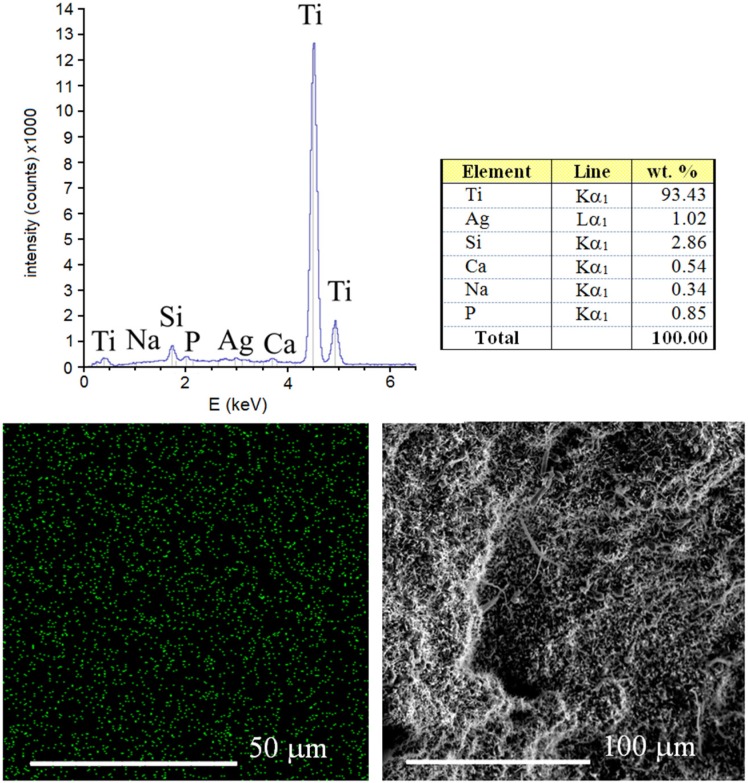
EDS surface spectrum and chemical composition analysis of the Ti-10 wt% 45S5 Bioglass-1 wt% Ag foam with distribution of Ag atoms.

The results of EDS analysis of the surface of sintered Ti-10 wt% 45S5 Bioglass-1 wt% Ag foam with 70% porosity is shown in Figure 5. It can be confirmed that the synthesized scaffold mainly consists of titanium matrix with elements of Na, Si, P, Ca and Ag. The benefit of the mechanical alloying technique application is possibility of sintering of materials with elements that are difficult or impossible to combine by conventional melting methods [30]. For example, calcium is soluble in titanium to the extent of at least 0.13 at% at 1300 °C [31].

**Figure 6:**
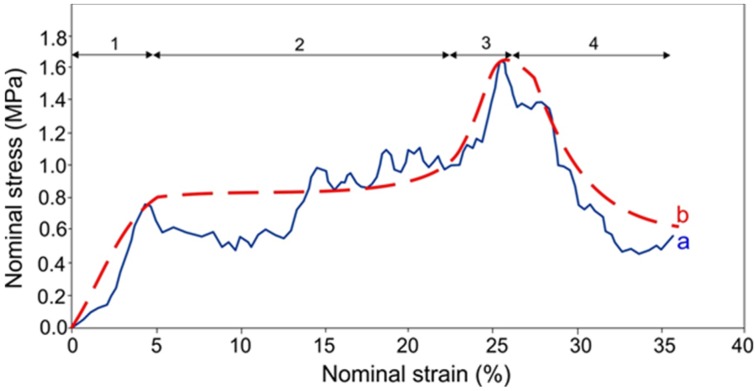
Stress-strain curves of the Ti-10 wt% 45S5 Bioglass-1 wt% Ag nanocomposite foam. Solid line—original curve (**a**) and dashed line of fitted curve (**b**); deformation stages are shown for fitted line (see description in text).

The Vickers’ hardness for bulk Ti-Bioglass-Ag composites reaches 480 HV_0.3_ and is higher than that of pure microcrystalline Ti metal (180 HV_0.3_). This effect is directly connected with structure refinement. Stress-strain curve recorded for Ti-Bioglass-Ag scaffold is shown in Figure 6. Due to high porosity, the sample is very brittle. Due to pore collapsing, scaffold cracking and its pulverization during sample compression, stress oscillations are visible and thus the typical plateau range is not flat and does not have a constant value (original curve a). The fitted stress-strain curve (b) shows typical behavior for scaffold of high porosity with wide plateau stress [32]. In this case the shape of the fitted stress-strain curve for metal scaffold shows four characteristic regions:

(1) elastic deformation—at initial raising strain (range 1 on Figure 6);
(2) stress plateau—the pores start to crash and the stress is almost the same with raising strain (range 2 on Figure 6);
(3) significant increase of stress related to pores being crushed and their collapse resulting in pore reduction and formation of bulk material (range 3 on Figure 6); and
(4) stress decrease due to destruction of scaffold is shown (range 4 on Figure 6).

The value of compression strength for the scaffold was 1.5 MPa, which is a typical value for highly porous foams [33]. The Young’s modulus was estimated to be 34 MPa. In comparison, the Young’s modulus of the cortical bone is 15–20 GPa and that of cancellous bone is 0.1–2 GPa. Compressive strength for cortical bone and cancellous bone is 100–200 MPa and 10–50 MPa, respectively [2]. The authors’ foams exhibit generally lower mechanical properties compared to bone; therefore, they need further porosity optimization. Moreover, the compressive strength is relatively low and is strictly related to the scaffold size and porosity of the foams.

**Figure 7:**
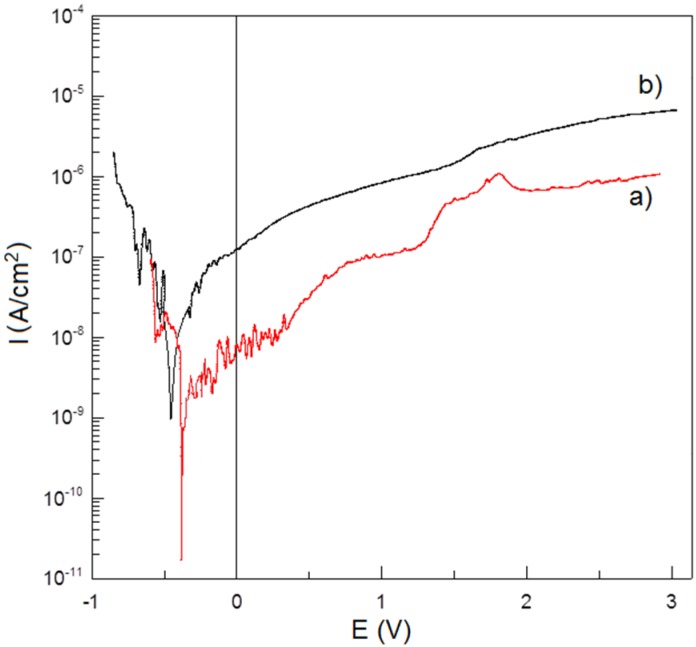
Potentiodynamic polarization curves of: Ti-10 wt% 45S5 Bioglass-1 wt% Ag nanocomposite foam with porosity of 70% (**a**) and microcrystalline titanium (**b**) in Ringer’s solution at 37 °C.

Corrosion resistance potentiodynamic test in Ringer’s solution at 37 °C shows no negative effect of porosity on Ti-Bioglass-Ag scaffolds in comparison to microcrystalline Ti (Figure 7). Titanium foam with 10 wt% 45S5 Bioglass and 1 wt% Ag has better corrosion resistance (*I*_C_ = 3.380 × 10^−9^ A/cm^2^, *E*_C_ = −0.377 V) than microcrystalline titanium (*I*_C_ = 1.089 × 10^−8^ A/cm^2^, *E*_C_ = −0.476 V). An enhanced corrosion resistance is due to rutile layer formed on the surface of Ti-Bioglass-Ag foam.

**Figure 8:**
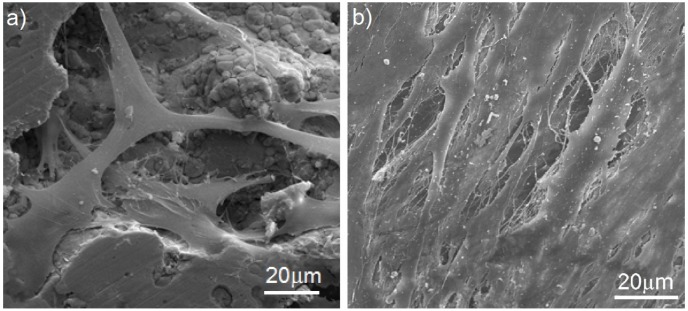
Scanning electron micrograph of osteoblasts cultured on the Ti-10 wt% 45S5 Bioglass-1 wt% Ag foam with a 70% porosity after one day (**a**) and seven days (**b**).

**Figure 9:**
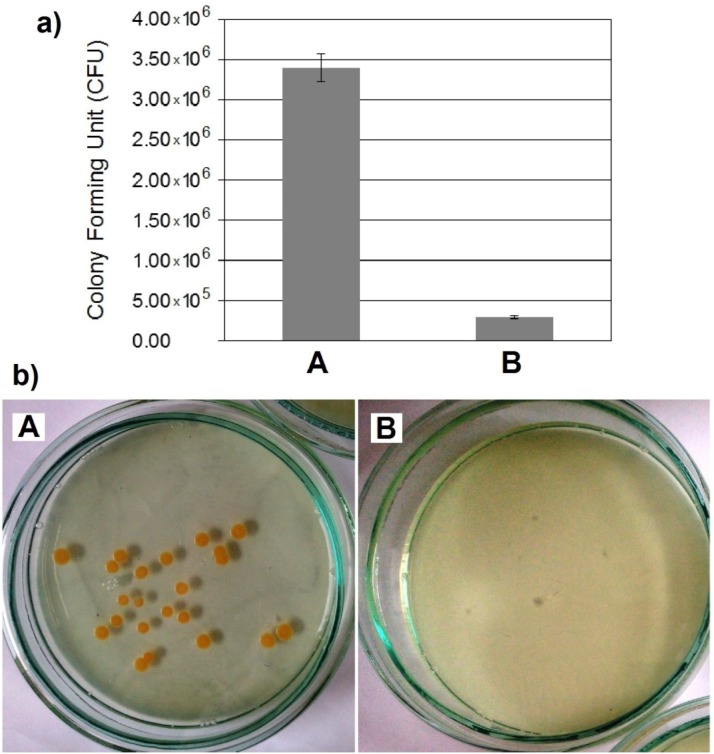
Statistical results of viable adherent bacteria on microcrystalline titanium (A) and bulk Ti-10 wt% 45S5 Bioglass-1 wt% Ag nanocomposite (B) (**a**) and representative macroscopic photos of viable adherent bacteria on difference experimental material surfaces (**b**).

The *in vitro* cytocompatibility test was conducted (Figure 8). Cell attachment and proliferation depended on the topography and roughness of the surface. The osteoblasts that grew on the inserts exhibited adhesion to the material surface after one day and covered the majority of the surface after seven days. Surface irregularities, such as pores, protrusions, and hillocks, enhance the stability of the cells on the implant surface. The inhibition of bacteria was investigated with the use of *S. aureus* (Figure 9). *In vitro* bacterial adhesion study indicated a significantly reduced number of *S. aureus* on Ti-45S5 Bioglass-Ag. The Ti-Bioglass-Ag composites were observed to have significantly lower adhesion of *S. aureus*, suggesting that the synthesized composites were antibacterial.

## 4. Discussion

Current goals in the development of new Ti-based biomaterials are: (i) to avoid potentially toxic elements, such as vanadium, to further improve biocompatibility; (ii) to produce titanium alloys with a high fatigue strength but a low Young’s modulus compared to cortical bone, to minimize stress shielding and improve fracture healing. Composite materials containing titanium and bioceramic as a reinforced phase are expected to have broad practical applications [20,34]. Our studies provided evidence of an enhancement of the properties due to the nanoscale structures in consolidated Ti or Ni-free 316L SS-HA composites [20,35]. Currently, the main challenge of tissue engineering is the development of an appropriate three-dimensional architecture of a scaffold serving cell adhesion, migration, growth, and proliferation [16].

In this work, the mechanical alloying and space-holder sintering processes were used to synthesize composite Ti-Bioglass-Ag foam. The corrosion test results indicated that the microcrystalline titanium possesses a lower corrosion resistance and consequently a higher corrosion current density (*I*_C_ = 1.089 × 10^−8^ A/cm^2^, *E*_c_ = −0.476 V) in the Ringer’s solutions. The Ti-Bioglass-Ag foam has a better corrosion resistance (*I*_C_ = 3.380 × 10^−9^ A/cm^2^, *E*_C_ = −0.377 V). The corrosion resistance is a very important property in implant applications because the corrosive environment of the tissue and body fluids may result in an unexpected local corrosion attack of the implant and the corrosion products may affect the tissue and the close vicinity of the implant, which results in poisoning of the tissue and surrounding environment.

Mechanical alloying technique allows alloying of elements that are difficult or impossible to combine by conventional melting methods. The high deformation during MA leads to the large dislocation density and formation of the nanometer size subgrains. These biomaterials possess unique mechanical and surface properties similar to the bone and hence are considered to be the future generation of biomaterials [16].

It is well known that the key factors for successful osseointegration are the surface properties of titanium implants. The surface structure of the sample directly effects the intensity of cell growth on it. For example, the porous surface, caused the cells to adhere with their entire surface to the insert as well as to penetrate the pores. Adhesion and growth on porous material is a specific characteristic of osteoblasts [36]. Burgers *et al.* proved that the pore size of approximately 200–500 µm is optimal for the attachment, differentiation and ingrowth of osteoblasts and moreover for vascularization [37]. However, recent reports show that also smaller micropores enhance living cell growth [16,38].

There are numerous reports that demonstrate that the surface roughness of titanium implants affects the biomechanical fixation [39,40]. Surface roughness can be divided into three levels depending on the scale of the features: macro-, micro-, and nano-sized topologies. The microtopographic profile of dental implants (in the range of 1–10 μm) maximizes the interlocking between mineralized bone and the surface of the implant [40]. On the other hand, surface profiles in the nanometer range play an important role in the adsorption of proteins, adhesion of osteoblastic cells and thus the rate of osseointegration [41]. The modification of the surface roughness of dental implants might hasten osseointegration. Further experiments are under investigation to dissociate more clearly surface topography and surface chemistry effects on human osteoblast proliferation and adhesion.

*In vitro* cytocompatibility tests of Ti-10 wt% 45S5 Bioglass nanocomposites and their scaffolds were performed [20]. Fibroblasts play a key role in the initial stage of the implant integration with surrounding tissue. The study proved that no cytotoxic properties of the samples have been seen, either on the tested nanomaterial sample or on microcrystalline one. However, the cell growth intensity is strongly dependent on the surface structure of the material. Obtained results show that nanocrystalline Ti-10 wt% 45S5 Bioglass sample displays good cytocompatibility compared to microcrystalline titanium. Survival rate of CCD-39Lu fibroblasts was higher in the presence of the nanocrystalline material as compared with microcrystalline material in the same concentrations of the same intervals. The proliferative activity of CCD-39Lu fibroblasts cultured in the presence of a nanocrystalline Ti-10 wt% 45S5 Bioglass material correlates with viability of these cells in the culture and is depended on the quantity of associated material.

The process of osseointegration takes place *in vitro* as well as *in vivo* in four stages [42,43]. In the first stage, stem cells and osteoblast precursor cells migrate to the implant and adhere to it by the formation of foot-like migration processes. In the second stage, the cells begin to be anchored onto the substrate wall with the help of matrix proteins and produce a dense network of matrix proteins at the implant. The mineralization of the extracellular matrix occurs in stage 3 and is dependent on differentiation of osteoblasts. In the last stage, the network of extracellular matrix proteins and osteoblasts reorganizes and forms lamellar bone [42].

The Ti-Bioglass-Ag composite showed the highest antibacterial activity against *S. aureus*. The biofilm formation was reduced by 91.3% in comparison to microcrystalline titanium and 99.0% to Ti-10 wt% 45S5 Bioglass composite. When Ti-Bioglass-Ag material is in contact with the body fluid, the metallic Ag particles at the surface of the material could go into a reaction with the fluid, leading to a release of ionized Ag into the surrounding. A steady and prolonged release of the silver biocide in a concentration level (0.1 ppb) is capable of rendering antibacterial efficacy [44,45]. Therefore the Ti-Bioglass-Ag composite can exhibit antibacterial activity, if there are enough Ag ions in the near surrounding.

The mechanism for bacterial toxicity of tested Ti-based nanocomposites may include not only free metal ion toxicity arising from the dissolution of metals from the surface of the silver particles (e.g., Ag+ from Ag) [46] but also oxidative stress via the generation of reactive oxygen species (ROS) on crystal surfaces of some nanoparticles (e.g., 45S5 Bioglass) [47]. Independent study proved that nanocrystalline Ti-10 wt% 45S5 Bioglass materials regardless to the form (powder or bulk disc) do not affect the viability of *Candida albicans* and do not inhibit the growth of yeast in direct or indirect contact [20]. Despite the fact that tested nanomaterials do not have negative influence against yeast, their structure and/or surface limits the growth of tested *Candida* yeast.

Published results clearly demonstrate that powder manufacturing routes produce Ti-based implants that are suitable for biomedical applications [48]. Due to the fact that the shape of the surface is often critical for tissue function, it seems that the composite Ti-Bioglass-Ag foam may be an important step towards the production of such a structure, which can support the process of adaptation of the implant by the host organism. More research is needed into the biocompatibility and functionality of these Ti-45S5 Bioglass-Ag foams.

## 5. Conclusions

A new kind of biomedical Ti-10 wt% 45S5 Bioglass-1 wt% Ag foam was prepared by mechanical alloying and powder metallurgy process. Saccharose crystals with the particle size 1 mm were used as the space-holder material. The following conclusions can be out drawn:

– the Ti-Bioglass-Ag foams with a relative density of approximately 0.3 were fabricated;
– foams with about 70% porosity have average pore diameter of about 0.3–1.1 mm in range, there were also micropores distributed on the macropore-edges with a size of several microns, which is expected to be beneficial to vascularization;
– the compression strength and Young’s modulus for the foam were 1.5 MPa and 34 MPa, respectively;
– the Ti-Bioglass-Ag foams are more corrosion resistant than the bulk microcrystalline titanium in Ringer solution;
– the cells on the Ti-Bioglass-Ag foam were observed to be well dispersed inside the pores; and
– the bulk Ti-Bioglass-Ag exhibits reduced bacteria adhesion (*S. aureus*) in comparison with microcrystalline titanium.

The main advantage of the study is to establish that the Ti-Bioglass-Ag composite foams can become an innovative material resource serving a new generation of medical implants.
